# Urachal adenocarcinoma in an adolescent girl: A case report

**DOI:** 10.1016/j.ijscr.2025.110846

**Published:** 2025-01-06

**Authors:** Elisamia Ngowi, Sonal Patel, Pilly Ally, Caroline Ngimba, Masawa Klint Nyamuryekung'e

**Affiliations:** a Department of Paediatrics and Child Health, Aga Khan Hospital Tanzania, P.O. Box 2289, Dar Es Salaam, Tanzania; b Department of Paediatrics and Child Health, Aga Khan University, P.O. Box 38129, Dar Es Salaam, Tanzania; c Department of Pathology, Aga Khan Hospital, P.O. Box 2289, Dar Es Salaam, Tanzania; d Department of Surgery, Aga Khan Hospital, P.O. Box 2289, Dar Es Salaam, Tanzania; e Department of Surgery, Aga Khan University, P.O. Box 38129, Dar Es Salaam, Tanzania; f Department of Radiology, Aga Khan Hospital Tanzania, P.O. Box 2289, Dar Es Salaam, Tanzania

**Keywords:** Urachus, Urachal adenocarcinoma, Adolescent, Case report

## Abstract

**Introduction:**

The urachus is a fetal canal that connects the allantois to the bladder and typically obliterates by the 6th month of gestation. Failure of the urachus to obliterate can result in urachal anomalies, which, in rare cases, may undergo malignant transformation.

Case presentation.

We present a case of a 13-year-old female who experienced hematuria, dysuria, and abdominal pain persisting for over 4 months. A CT scan revealed a mass extending from the bladder wall, involving an adjacent bowel loop, and associated with intra-abdominal lymphadenopathy. Debulking surgery was performed, and a histopathological examination confirmed the diagnosis of urachal adenocarcinoma.

**Discussion:**

Urachal anomalies are exceedingly rare, with malignancies arising from urachal remnants being even more uncommon. Most patients are diagnosed at advanced stages due to the late onset of symptoms, resulting in a five-year survival rate of approximately 50 %.

**Conclusion:**

Urachal adenocarcinoma can occur in children, potentially due to early oncogenesis of urachal cells. It should be considered a significant differential diagnosis in children presenting with recurrent lower abdominal pain and a urachal remnant to facilitate early detection and timely management.

## Highlights

• Urachal malignancies are rare and differentiate from urachal remnants.
• Most urachal malignancies occur after the 5th decade of life.
• Hematuria, dysuria and abdominal pain are commonest symptoms encountered.
• Most cases are diagnosed in late stages, hence poor outcomes in these patients.

## 1 Introduction

The urachus is a canal approximately 5 to 10 cm in length that connects the allantois to the fetal bladder during early gestation. It consists of three distinct layers: a luminal layer of cuboidal or transitional epithelium, an intermediate submucosal connective tissue layer, and an outer layer of smooth muscle. Under normal development, the urachus obliterates into a fibromuscular cord extending from the umbilicus to the bladder dome during the fourth and fifth months of fetal development. Failure to this obliteration can result in urachal anomalies, though the occurrence of urachal malignancies remains exceptionally rare [1,2].

Urachal carcinomas constitute <1 % of all bladder malignancies and have a higher incidence in males compared to females. Most reported cases occur in adults during their fifth to sixth decade of life, making the condition uncommon in children. Among urachal carcinomas, adenocarcinomas are the most prevalent subtype. These cancers are associated with a poor prognosis, primarily due to delayed presentation and diagnosis. The estimated five-year survival rate for patients with urachal carcinoma is approximately 50 % [[1], [2], [3], [4]].

The most common presenting symptoms of urachal adenocarcinoma include hematuria, dysuria, and abdominal pain. In some cases, a palpable abdominal mass is detected, which may be the sole presenting sign [1,5].

The disease can be staged using either the Shelson or Mayo staging systems, both of which have been shown to effectively predict patient prognosis. For nonmetastatic disease, surgery remains the recommended treatment option, with organ-preserving partial cystectomy being the preferred approach [4].

We present a case of an adolescent girl who was diagnosed with urachal adenocarcinoma, a very rare bladder malignancy in children.

This case report has been reported in line with the SCARE criteria [6].

## 2 Case presentation

We present the case of a 13-year-old female who had a four-year history of chronic abdominal pain. Her symptoms worsened over time, and she presented with lower abdominal pain, new-onset dysuria, terminal hematuria, and purulent mucoid discharge. She also reported a progressively enlarging lower abdominal mass over the past six months, accompanied by gradual weight loss and intermittent fever spikes. Notably, she had not yet attained menarche and showed no features of precocious puberty. A review of other systems revealed episodes of headache, myalgias, and palpitations. Before presenting at our facility, she had been treated for suspected extrapulmonary tuberculosis without improvement over five months at another healthcare center.

The patient's early childhood history was unremarkable, with no prior surgeries or chronic illnesses.

On examination, she appeared pale, cachectic, and tachycardic. An abdominal assessment revealed a scaphoid abdomen with a palpable hard mass extending from the hypogastric region to the right iliac region. The mass was ill-defined, firm, tender, and had a rough surface. It extended into the pelvis, demonstrating a dull percussion note, and showed no evidence of shifting dullness.

The clinical impression suggested a locally advanced neoplastic process, possibly originating from the bowel, ovary, or uterus, with potential secondary or primary involvement of the bladder, contributing to the irritative urinary symptoms, cachexia, and anemia indicative of advanced disease. Infectious etiologies, such as Human Immunodeficiency virus (HIV) infection, were also considered as differential diagnosis.

Laboratory investigations showed severe anemia with hemoglobin levels of 6.3 g/dL (range; 12.3–15.3 g/dL), high total white blood cell count of 15.4 × 10^9^ (normal; <16 × 10^9^), elevated C-reactive protein of 142 mg/L (normal; 0.5–5), serological test for Human Immune deficiency viruses 1 and 2 were negative. Serum Beta HCG was <0.1 mIU/mL (normal; <5.3 mIU/mL). Serum CA125 was 32.45 U/mL (normal; <35 U/mL). Urine analysis was positive for nitrites and leucocytes indicating urinary tract infection.

**Fig. 1:**
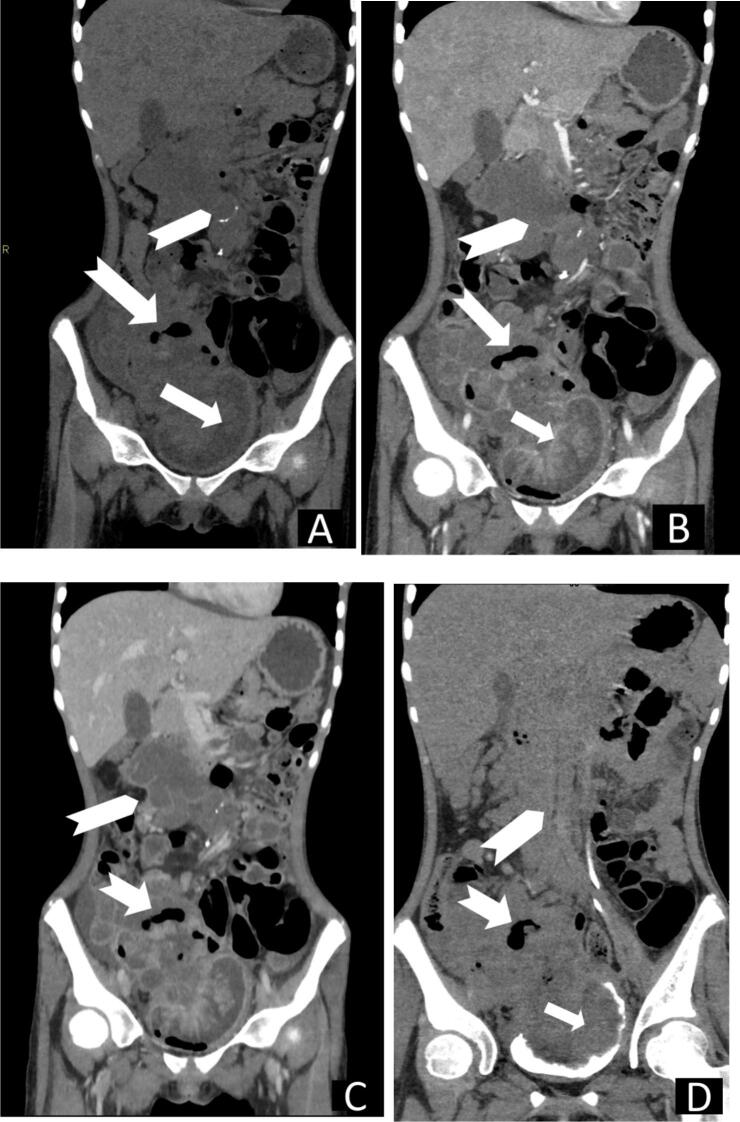
Computed tomography (CT), coronal reconstructed images (**A**) pre-contrast, (**B**) arterial phase, (**C**) portal venous phase and (**D**) delayed images revealed a right lower abdominal mass (**arrows**) extending from the urinary bladder through the right superior and lateral wall, breaching the bladder wall and almost filling the lumen. There is associated distal ileal loop inflammation, fistulous formation between bladder and bowel loops and air-filled cavity within the mass (**notched white arrows**), and intraabdominal adenopathy and calcification (**white chevron**).

**Fig. 2:**
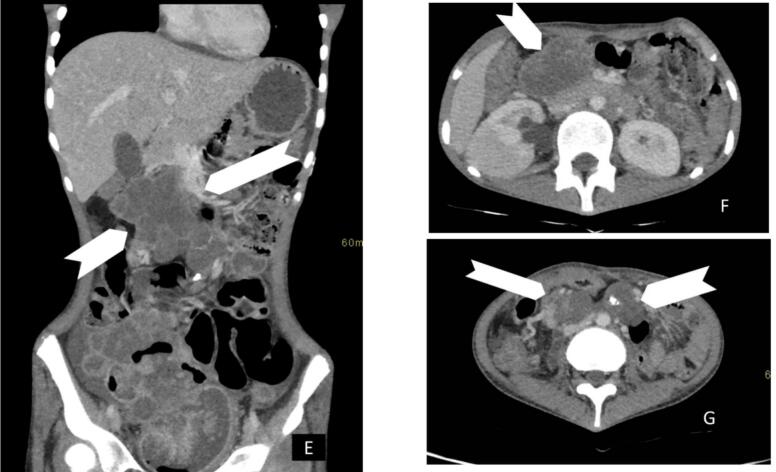
Computed tomography Images, (**E**) coronal, (**F**) axial images at the level of the kidneys, and (**G**) axial image at the level of the umbilicus demonstrating enlarged lymph nodes with calcification (**white chevron**).

A computed tomography (CT) scan revealed an abdominal mass originating from the urinary bladder, breaching the bladder wall, and extending to the distal ileal loop, causing inflammation and forming a fistula. Additionally, the scan showed abdominal lymphadenopathy and calcification as seen in Fig. 1, Fig. 2.

Given the above presentation, she was admitted and given broad-spectrum antibiotics to treat the urinary tract infection. A multidisciplinary discussion was held, and a decision was made for Cystoscopy and biopsy for histology of the neoplasm; intraoperatively a right-sided ventrolateral polyploid tumor with a vesicoureteral fistula was encountered, sparing the trigone, ureteric orifices, and about 60–70 % of the bladder. A biopsy was taken for histology study.

**Fig. 3:**
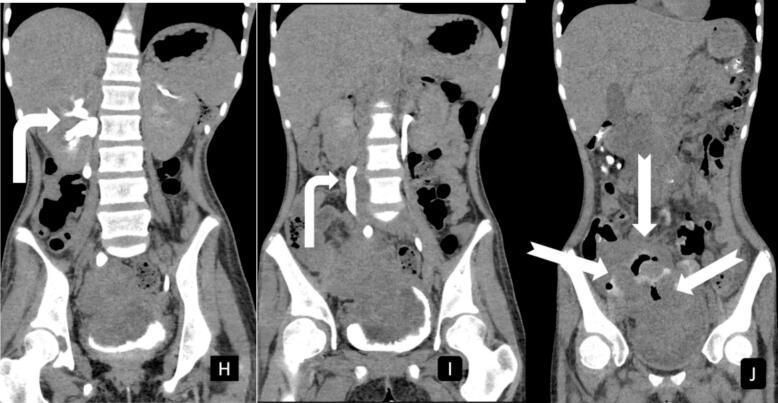
Coronal reconstructed delayed images post-IV contrast show mild hydronephrosis with ipsilateral hydro ureter image (**H**) and (**I**) **white bent arrows**. The ureter could be traced down to just above the iliac crest level where there is a large mass-like lesion of matted bowel loops appearing to be extending into the urinary bladder lumen via a breech at the fundus of the bladder. Air is seen within the bladder lumen. Post-oral contrast the images confirm the presence of fistulous communication between bladder and bowel loops as well as bowel loops and the focal air collection within the matted structures image (**J**) **notched arrows**.

The partial right ureteric obstruction, with hydroureteronephrosis, is demonstrated on CT scan imaging, as in Fig. 3.

Histology of the biopsy showed fragments of tissue with a moderately differentiated adenocarcinoma. Immunohistochemistry on it was strong and diffusely positive for CK (cytokeratin) 20, CDX2, and focal positivity for CK (cytokeratin) 7. GATA 3 was positive in the residual urothelium. These findings were in keeping with urachal adenocarcinoma.

**Fig. 4:**
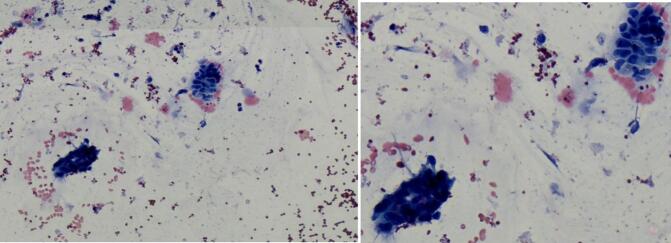
Cytology of the ascetic fluid showed haemorrhagic background and clusters of atypical hyperchromatic cells forming glandular/ductal structures.

To assess resectability, a diagnostic laparoscopy was performed with camera placed through the umbilicus. The findings revealed a right-sided pelvic polyploid solid lesion extending from the urachal remnant to the bladder. The lesion was mobile and involved the small bowel loop and the right adnexa. No signs of small or large bowel dilatation were observed, and no obvious carcinomatosis was present. A minimal amount of serosanguinous ascitic fluid was noted and collected for cytological analysis, as shown on Fig. 4, which corresponds to Stage IV under the Mayo Staging system.

**Fig. 5:**
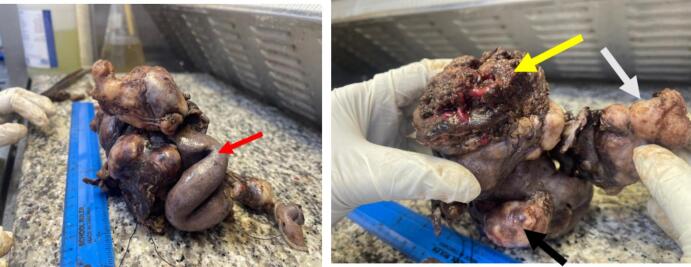
Gross sections of the complete resected tumor which included partial cystectomy, segmental bowel resection, and right salpingo-opherectomy. (**Red arrow**) - bowel, (**yellow arrow**) - urinary bladder, (**white arrow**) - tumor, and (**black arrow**) - ovary. The cut section of the tumor was solid to cystic in consistency, with mucoid material. It involved the urinary bladder and ovary. (For interpretation of the references to color in this figure legend, the reader is referred to the web version of this article.)

Given that surgery was the most effective treatment for urachal adenocarcinoma, and no established chemotherapeutic regimens were available, a multidisciplinary team meeting recommended proceeding with complete tumor excision. This would involve partial cystectomy, segmental bowel resection, and right salpingo-oophorectomy, should the adnexa be found to be involved. The urachal remnant and median umbilical ligaments were excised, segmental ileal resection, partial cystectomy with a 1 cm margin, and right salpingo-opherectomy was done, as seen in Fig. 5. The urachus and umbilicus were excised en bloc with the tumor. Bladder reconstruction was performed using an end-to-end ileal-ascending colon anastomosis during the same surgical procedure. Enlarged retroperitoneal lymph nodes, including the internal iliac and para-aortic lymph nodes, measuring approximately 3 by 4 cm, were encountered intraoperatively. These nodes were fixed to the major vessels, and dissection was not feasible due to the absence of a clear surgical plane between the lymph nodes and the vessels.

Postoperatively, the patient was placed on antibiotics, provided with appropriate pain management, and given adequate bladder irrigation. A multidisciplinary team, including dietitians and physiotherapists, was involved in her care to optimize recovery. The patient was subsequently discharged and scheduled for follow-up at the clinic.

**Fig. 6:**
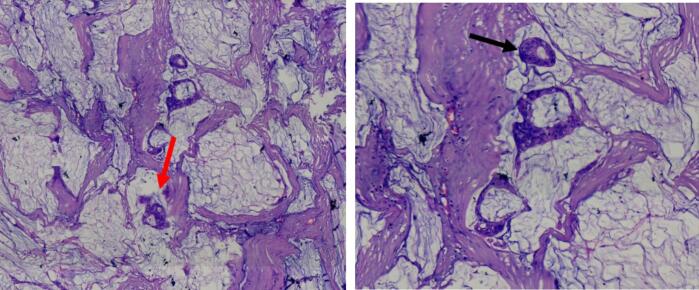
Histopathology of the resected specimen showed a malignant neoplasm predominantly showing neoplastic glands floating on mucin pools (**red arrow**). Other sections showed moderately differentiated glands (**black arrow**). (For interpretation of the references to colour in this figure legend, the reader is referred to the web version of this article.)

The post-operative histopathology showed neoplastic glands on mucin pools as in Fig. 6, and post operative staging was stage 4.

She was then planned for adjuvant chemotherapy due to the increasing size of the abdominal lymph nodes at 6 weeks post-surgery which were suspicious for disease progression. She was initiated on a 4-cycle regimen of intravenous Gemcitabine at a dose of 1000 mg/m^2^ and intravenous Cisplatin at a dose of 75 mg/m^2^. At the follow-up clinic, the patient reported no new symptoms and continued with chemotherapy. However, the prognosis remains guarded, as there are limited management protocols in the literature due to the rarity of this malignancy.

## 3 Discussion

Urachal anomalies are exceedingly rare and are often diagnosed incidentally during ultrasound examinations. Most urachal anomalies and remnants contain epithelial components, which serve as a major risk factor for the development of adenocarcinoma later in life [7]. Since urachal anomalies are often asymptomatic, malignant changes are typically present only in the advanced stages, usually after the fifth decade of life. The incidence of such malignancies is higher in males than in females [8]. Our patient was female and presented with symptoms during adolescence, which is atypical for this malignancy, as it is rarely observed in this age group.

The most frequent clinical manifestations of urachal adenocarcinomas, as reported in various adult cases, include hematuria, abdominal pain, and dysuria. Palpable masses are typically felt in the suprapubic region. The classic presentation may also involve the discharge of pus or blood from the umbilicus [1,9]. Our patient presented with symptoms consistent with urachal adenocarcinoma, including abdominal pain, dysuria, and hematuria. However, there was no history or evidence of umbilical discharge, even during her early childhood.

Due to limited research on urachal carcinomas, there is no established consensus on the most appropriate biological markers to investigate. However, elevated levels of certain tumor markers, particularly carcinoembryonic antigen (CEA), A-125, CA 19-9, and CA 72-4, have been reported in many patients with urachal cancers. These markers are considered important predictors of therapeutic response [10]. Our patient had normal levels of CA125 but did not test for other immunohistochemical biomarkers.

The diagnosis of urachal carcinoma is based on a combination of medical history, physical examination, and radiological imaging, with cystoscopy and biopsy used to confirm the cellular type. Cystoscopy is a crucial diagnostic tool, yielding positive results in approximately 90 % of cases, and is instrumental in identifying tumor location, typically at the bladder dome or anterior wall. Ultrasonography is useful for visualizing the mass and assessing its consistency. A midline supravesical soft tissue mass identified on ultrasound or CT scan is highly suggestive of urachal abnormalities. CT scans and MRIs are valuable for detecting calcifications in mucin-producing adenocarcinomas, although calcifications are not present in all cases. Additionally, CT and MRI provide critical information about the extent of metastasis and lymph node involvement [4]. Our patient underwent a cystoscopy and a CT scan of the abdomen and pelvis, which were instrumental in guiding the debulking surgery. Ultrasonography was not performed, as the CT scan provided sufficient detail to visualize the extent of the tumor and detect metastatic lesions.

Recent advancements in DNA sequencing technologies, such as next-generation sequencing, have facilitated the differentiation between urachal adenocarcinoma and bladder adenocarcinoma. This novel approach has revealed that urachal adenocarcinoma is associated with specific gene mutations, including RAS, GNAS, SMAD4, and BRAF, which are absent in bladder adenocarcinoma. Among these, SMAD4 mutations show a stronger association with the mucinous subtype of urachal adenocarcinoma compared to other histological types [11,12]. In low-resource settings, performing genetic studies for diagnostic purposes may not be feasible due to the unavailability of advanced technology and the high costs associated with such tests. Additionally, logistical challenges, such as transporting specimens to laboratories in foreign countries, further complicate the process. Genetic studies were not conducted in our patient's case, primarily due to the limited resources available in this setting.

Treatment of urachal adenocarcinomas typically involves a combination of surgical excision, radiotherapy, and chemotherapy. Surgical intervention remains the most effective management strategy, with partial or total cystectomy and en bloc excision of the urachal mass being the standard procedures and achieving local disease control is crucial for treatment success. There is approximately a 40 % risk of disease recurrence in urachal carcinomas, whereas recurrence is significantly lower in urachal sarcomas. Local recurrence may result from tumor seeding within the distal urothelial tract, particularly in polypoid tumors that have breached the bladder cavity. Adjuvant therapy, including chemotherapy or radiotherapy, is considered for patients with positive lymph nodes or surgical margins to improve outcomes [1,2,13]. In our case, total resection of the tumor was planned; however, intraoperatively, retroperitoneal lymphadenectomy could not be performed due to technical challenges. Approximately one month after surgery, the patient presented with an increase in the size of the lymph nodes, suggesting disease progression.

Several chemotherapeutic agents have been evaluated in studies for the management of urachal adenocarcinomas. Cisplatin-based combinations are considered the first-line treatment options, including regimes such as methotrexate, vinblastine, doxorubicin, and cisplatin (MVAC) or gemcitabine-cisplatin [4]. In our case, the patient was treated with cisplatin and gemcitabine.

Studies indicate a 50 % five-year survival rate following the diagnosis of urachal carcinoma. Tumor grade and the status of surgical margins are recognized as independent predictors of survival outcomes in these cases. The first staging system was introduced by Shelson et al. in 2984. Since then, several novel staging systems have been proposed, including the Mayo and Ontario staging systems. Most recently, the TNM staging system proposed by Limonnik et al. has been introduced as a comprehensive approach to staging urachal carcinomas [1,2,14]. According to these various staging systems, our patient presents with regional lymph node involvement and tumor invasion of the pelvic organs, which would classify her as stage 4.

## 4 Conclusions

This case highlights that urachal adenocarcinoma can occur at younger ages, suggesting that urachal remnants may transform into malignant cells more rapidly than typically observed. While risk factors remain unclear, physicians must consider urachal remnants in younger children presenting with unexplained chronic abdominal pain. Regular follow-up is essential for patients with persistent asymptomatic urachal remnants beyond early childhood, as early detection and intervention may improve outcomes.

## Ethical approval

Not required for case reports at our hospital for single case reports.

## Guarantor

Elisamia Ngowi, is the main guarantor of this research work. Elingowi80@gmail.com.

## Consent for publication

Written informed consent was obtained from the mother for the publication of this case report and the accompanying images. A copy of the written consent is available for review by the corresponding author of this journal.

## Declaration of Generative AI and AI-assisted technologies in the writing process

During the preparation of this work, the author(s) used Grammarly to correct sentence grammar and spelling. After using this tool/service, the author(s) reviewed and edited the content as needed and take(s) full responsibility for the content of the publication.

## Funding

No funds were needed to publish this case.

## Authors' contributions

All authors read and approved the final manuscript.

## Declaration of competing interest

The authors declare that they have no competing interests.

## Data Availability

The datasets of the present study are available from the corresponding author upon request.
